# The Influence of Radical Prostatectomy on the Expression of Cell-Free MiRNA

**DOI:** 10.3390/diagnostics10080600

**Published:** 2020-08-17

**Authors:** Maria Yu. Konoshenko, Olga E. Bryzgunova, Evgeniy A. Lekchnov, Evgeniya V. Amelina, Sergey V. Yarmoschuk, Svetlana V. Pak, Pavel P. Laktionov

**Affiliations:** 1 E.N. Meshalkin National Medical Research Center of the Ministry of Health of the Russian Federation, Novosibirsk 630055, Russia; olga.bryzgunova@niboch.nsc.ru (O.E.B.); lekchnov@gmail.com (E.A.L.); s_jarmoschuk@meshalkin.ru (S.V.Y.); s_pak@meshalkin.ru (S.V.P.); lakt@niboch.nsc.ru (P.P.L.); 2 Institute of Chemical Biology and Fundamental Medicine SB RAS, Novosibirsk 630090, Russia; 3 The Center for Technology Transfer and Commercialization, Novosibirsk State University, Novosibirsk 630090, Russia; amelina.evgenia@gmail.com

**Keywords:** cancer, prostate cancer, miRNA, cell-free miRNA, extracellular vesicles, radical prostatectomy, urine, blood plasma

## Abstract

MiRNAs of blood and urine have been shown to represent a convenient source of biomarkers for prostate cancer (PCa) diagnosis and assessment of the therapy effectiveness due to their high stability and representation and the low invasiveness of sample collection. Here, we studied the influence of radical prostatectomy (RP) on the expression of 12 cell-free miRNAs previously shown as potential markers of PCa (i.e., miR-19b, miR-22, miR-92a, miR-378, miR-425, miR-30e, miR-31, miR-125b, miR-200b, miR-205, miR-375 and miR-660). The relative expression of the miRNAs combined into 31 paired ratios was evaluated in the urine extracellular vesicles (EVs), clarified urine (CU) and blood plasma of healthy donors, pre- and post-RP samples of PCa patients. Nineteen miRNA ratios based on combinations of ten of the miRNAs (miR-19b, miR-30e, miR-31, miR-125b, miR-200b, miR-205, miR-375, miR-378, miR-425, and miR-660) were altered by RP. The comparative expression analysis of the cell-free miRNA ratios between healthy donors and PCa patients revealed miR-125b/miR-30e and miR-375/miR-30e as potential markers for evaluating therapeutic efficacy. MiR-378/miR-19b, miR-425/miR-19b, miR-200/miR-30e, miR-660/miR-30e, and miR-205/miR-30e had minor prognostic value but could be used to increase the steadiness of the diagnostic system. The urine EVs had the highest potential as a source of markers.

## 1. Introduction

Prostate cancer (PCa) is the second most common cancer worldwide and the fifth most common cause of cancer-related death among men. The most accepted therapy of localized PCa is radical prostatectomy (RP). Despite the development of PCa diagnosis and the improvement of surgical technique, local relapse after surgery remains an urgent problem. Biochemical recurrence occurs in 20–40% of PCa patients undergoing RP [1]. A substantial challenge in PCa research is to develop effective predictors of tumor recurrence following surgery to determine whether immediate adjuvant therapy is warranted. The serum PSA (prostate serum antigen) level and density, pathologic anatomic stage, Gleason score, nature of the surgical margin, tumor volume, and lymphovascular and perineural invasion are used in clinical practice for PCa relapse prediction. Nevertheless, the frequency of PCa relapses after RP is still high, indicating the necessity for the development of unified and reliable markers for monitoring the effectiveness of therapy and for predicting PCa relapse that would thus enable to select efficient treatment tactics.

One of the most modern promising sources of diagnostic and prognostic markers is a pool of cell-free miRNA, which are characterized by high stability and representation in biologic fluids. They may appear in the biofluids mainly through apoptosis, necrosis, oncosis, inflammation and active secretion (as a part of exosomes or microvesicles); in the urine, they may appear both from the blood and from the cells of the urogenital system [2,3]. Along with the presence of tumor-specific miRNAs in postoperative tissues (e.g., [4,5,6]), miRNAs of tumor origin have been found in biologic fluids, such as blood and urine, which seem to be a more promising source of materials for PCa diagnostic applications. Monitoring of cell-free miRNA levels in biologic fluids allows for minimally invasive procedures well-suited for periodical patient care in the long term (not just at the time of RP) and for surveillance of patients after different types of PCa therapy, such as hormonal or radiation therapy. However, only a few studies aimed at examining whether circulating miRNAs associated with PCa change from before to after RP are known to date [7,8,9]. It should be noted that different mechanisms are responsible for cell-free miRNA packaging and secretion, such as membrane covered extracellular vesicles (EVs) or nucleoprotein complexes (NPCs) [10]. Thus, miRNA content in EVs and NPCs differ [11], but both of these miRNA pools can be promising sources of cancer-related miRNAs [12,13]. Summarizing the above, the study of cell-free miRNA expression in the EVs and NPCs of PCa patients appears to be a reasonable approach for the identification of prognostic and therapeutic efficacy markers.

Earlier, we demonstrated that, selected from 84 miRNAs [14], a panel of 12 cell-free miRNAs—namely, miR-19b, miR-22, miR-92a, miR-378, miR-425, miR-30e, miR-31, miR-125b, miR-200b, miR-205, miR-375 and miR-660—allows us to classify patients with PCa and benign prostate hyperplasia (BPH) against healthy donors (HD) with 100% specificity and 100% sensitivity [15].

The aim of the present work was to select the minimum set of miRNA-markers and to evaluate a sample size met for statistical significance testing with high significance level and power, as well as to identify an optimal cell-free miRNA source for the next validation stage based on the executed study of preselected cell-free miRNA expression in the blood plasma, clarified urine (NPCs+EVs) and urine EVs of HDs and PCa patients before and after RP.

## 2. Materials and Methods

### 2.1. Sample Collection

Blood and urine samples from 11 healthy males and 10 PCa patients were obtained from E. Meshalkin National Medical Research Center of the Ministry of Health of the Russian Federation (Novosibirsk, Russia). The biofluid samples of the PCa patients were collected twice—once before and once after RP. The median follow-up time after RP was 6 days (Q_1_ = 5; Q_3_ = 9). The age range and mean age, the blood PSA, the disease stage and the Gleason score (for PCa patients) of the study population are shown in Table 1. This work was conducted in compliance with the principles of voluntariness and confidentiality in accordance with the “Fundamentals of Legislation on Health Care”, and was approved by ethical committees of ICBFM SB RAS (N 15309-01 from 22.12.2008). Written informed consent was provided by all participants.

Venous blood was collected in EDTA (ethylenediaminetetraacetic acid) spray-coated vacutainers, stored at 4 °C and processed within 4 h. Blood was sequentially centrifuged at 400× *g* for 20 min and 800× *g* for 20 min, both at 4 °C, to prepare the blood plasma. To remove cellular debris, the samples were centrifuged at 17,000× *g* at 4 °C for 20 min.

Fresh urine samples were collected in sterile containers. Urinary cells and debris were removed by sequential centrifugation at 400× *g* for 20 min at room temperature and clarified at 17,000× *g* for 20 min at 24 °C to obtain the clarified urine.

### 2.2. Isolation of Urine EVs by Ultracentrifugation

A volume of 5 mL of human urine (after clarified at 17,000× *g*) was diluted to 12 mL in phosphate-buffered saline (PBS), transferred to a 14-mL open-top Ultra-Clear^TM^ centrifuge tube (Beckman Coulter, Brea, CA, USA) and centrifuged at 100,000× *g* for 90 min at 18 °C in a Beckman Coulter Optima TM L-90k centrifuge with an Sw40Ti rotor (Beckman Coulter). The pellet was washed by resuspending it in 10 mL of PBS and pelleting it under the same conditions. Finally, the pellet was resuspended in 500 µL PBS, snap-frozen in liquid nitrogen and stored at −80 °C.

### 2.3. Isolation of miRNAs by the Gu/OcA Protocol

Before isolation of the miRNAs, the blood plasma or urine samples were thawed and gently mixed. Gu/OcA miRNA isolation (using guanidine thiocyanate and octanoic acid) from the urine and blood plasma was performed as described previously by Lekchnov et al. [16]. Isolation from urine EVs was performed as described for clarified urine. After the addition of denaturation buffer, the synthetic cel-miR-39-3p spike-in was added to the samples at 5 × 10^7^ copies per isolation.

### 2.4. RNA Precipitation

RNA precipitation by isopropanol was performed as described previously in [16]. To stabilize the miRNAs, 1.5 µL of glycogen (20 mg/mL) was added into each tube. Air-dried miRNA pellets were dissolved in 30 µL of RNAse-free water.

### 2.5. Reverse Transcription and Quantitative RT–PCR

Primers and probes for reverse transcription and TaqMan qPCR [15] were synthesized in the Laboratory of Medicinal Chemistry (ICBFM SB RAS, Novosibirsk, Russia), their sequences are presented in Table S1. Each reverse transcription (RT) reaction was performed in a total volume of 10 μL and contained 2.5 μL of RNA, 25-nM of each of the miRNA-specific primers, 0.5 units of RiboLockRNAse inhibitor (Fermentas, Vilnius, Lithuania), 50 units of M-MuLV–RH reverse transcriptase (BiolabMix, Novosibirsk, Russia), 2 μL of 5 × MMLV reaction buffer (250-mM Tris-HCl (pH 8.3 at 25 °C), 250-mM KCl, 20-mM MgCl_2_ and 50-mM DTT) and 125-mM of each dNTP. The reaction conditions were as follows: 16 °C for 30 min, 42 °C for 30 min and 70 °C for 10 min. Samples without RNA templates were used as negative controls. Real-time PCR was carried out on the CFX 96TM real-time system (Bio-Rad, Hercules, California 94547, USA). All reactions were carried out in duplicate in a total volume of 24 μL. Each reaction contained 4 μL of RT product, 1 unit of Taq DNA polymerase (BiolabMix, Russia), 2.4 μL of 10 × PCR buffer (750-mM TrisHCl (pH 8.8 at 25 °C), 200-mM (NH_4_)_2_SO_4_ and 0.1% (*v/v*) Tween-20), 3.2-mM MgCl_2_, 200-mM of each dNTP, 480-nM miRNA-specific forward primer, 640-nM universal reverse primer and 240-nM of the specific TaqMan probe [13]. After an initial denaturation at 95 °C for 3 min, the reactions were run for 50 cycles at 95 °C for 15 s and 60 °C for 45 s. The threshold cycle (Ct) values of the assessed miRNAs were compared between samples from different donor groups. The miRNA expression was evaluated in two sets—i.e., miR-19b, miR-22, miR-92a, miR-378, miR-425 and cel-miR-39 and miR-30e, miR-31, miR-125b, miR-200b, miR-205, miR-375 and miR-660.

### 2.6. Statistical Analysis

Statistical analysis was carried out with Statistica software 6.0. The Ct values were used to perform ratio-based normalization, effectively evaluating the relative expression of all possible combinations of any two miRNAs in the sample [17,18]. Because miRNA expression was evaluated in two sets of five and seven miRNAs, normalization was only used within each group. Thus, 31 miRNA ratios were formed from the 12 analyzed miRNAs. For every ratio, the Ct difference (dCt) values and the differences in the miRNA ratio levels before and after RP (ddCt) were calculated. For each miRNA pair, the mean dCt and ddCt values were calculated in each of the studied fractions of biologic fluid. Comparisons between PCa and HDs were done using one-way analysis of variance (ANOVA), followed by Fisher’s post-hoc test. The comparison between PCa patients before and after RP was performed with Wilcoxon signed-rank test. A *p*-value of <0.05 was considered statistically significant. Benjamini–Hochberg correction (p_adj_) was used to adjust the statistical significance for multiple comparisons. The sample size calculation was carried out using R (library pwr) to ensure the minimum threshold was met for statistical significance testing with determined significance level (5%) and power (80%). We used “one-sample” and “two–sided” versions to estimate the necessary sample size for establishing a treatment effect (the difference in dCt values before and after RP). The effect size was calculated using the mean difference between the dCt values of each miRNA pair before and after RP. The standard deviation estimations were calculated using the available samples.

## 3. Results

### 3.1. The Influence of the RP on the Expression of Cell-Free miRNA

**Figure 1:**
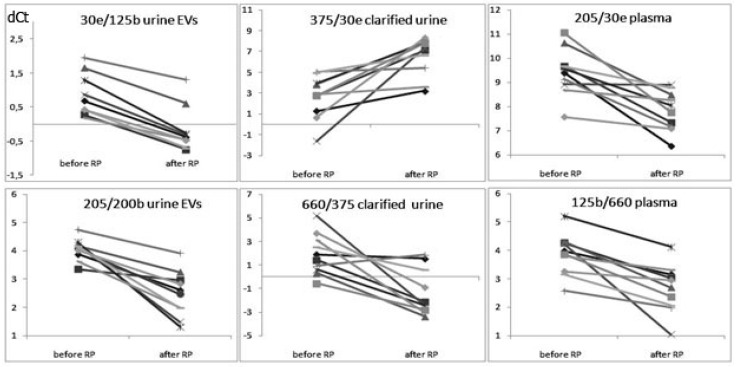
Examples of the miRNA ratios (dCt) whose expression was altered after RP of prostate cancer (PCa) patients. Wilcoxon signed-rank test, *p* < 0.01. EVs—extracellular vesicles; RP—radical prostatectomy. Different colored lines represent different donors.

**Figure 2:**
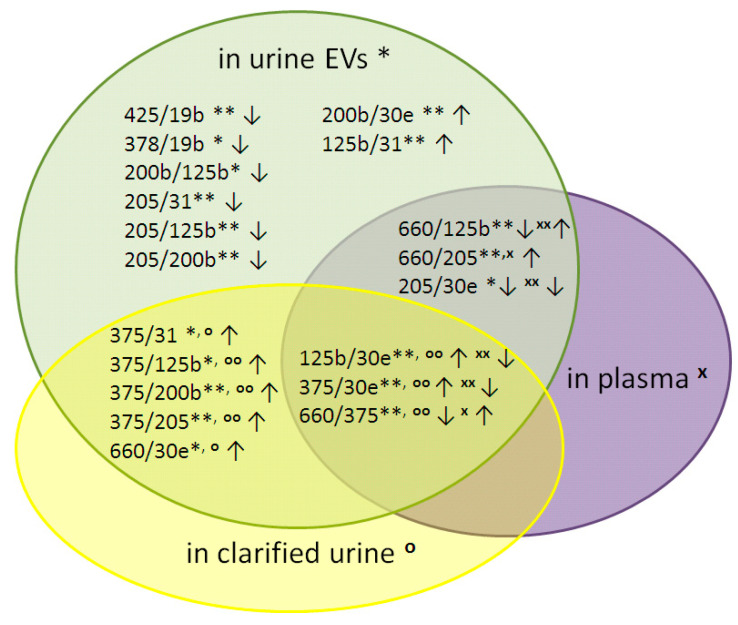
Euler–Venn diagram of the relative expression of the miRNAs in urine EVs, clarified urine (CU) and blood plasma. Arrows represent the ratio of the relative expression of the miRNA pairs before and after RP. Wilcoxon signed-rank test for different samples: Urine EVs, ** *p* < 0.01 and * *p* < 0.05; CU, ^oo^ *p* < 0.01 and ^o^ *p* < 0.05; blood plasma, ^xx^ *p* < 0.01 and ^x^ *p* < 0.05.

The comparison between PCa patients before and after RP was performed in paired samples from each PCa patient using Wilcoxon signed-rank test. The expression of 19 miRNA ratios (based on ten different miRNAs)—namely, miR-378/miR-19b, miR-425/miR-19b, miR-200b/miR-30e, miR-200b/miR-125b, miR-205/miR-31, miR-205/miR-125b, miR-205/miR-200b, miR-125b/miR-31, miR-375/miR-31, miR-375/miR-125b, miR-375/miR-200b, miR-375/miR-205, miR-660/miR-30e, miR-125b/miR-30e, miR-375/miR-30e, miR-660/miR-375, miR-660/miR-125b, miR-660/miR-205 and miR-205/miR-30e—was significantly altered after RP in at least one fraction of the biologic fluids of PCa patients. Among these altered ratios, 19 were detected in urine EVs, 8 in CU and 6 in blood plasma (Figure 1 and Figure 2; Table 2). All ddCt ratios are presented in Table S2.

**Figure 3:**
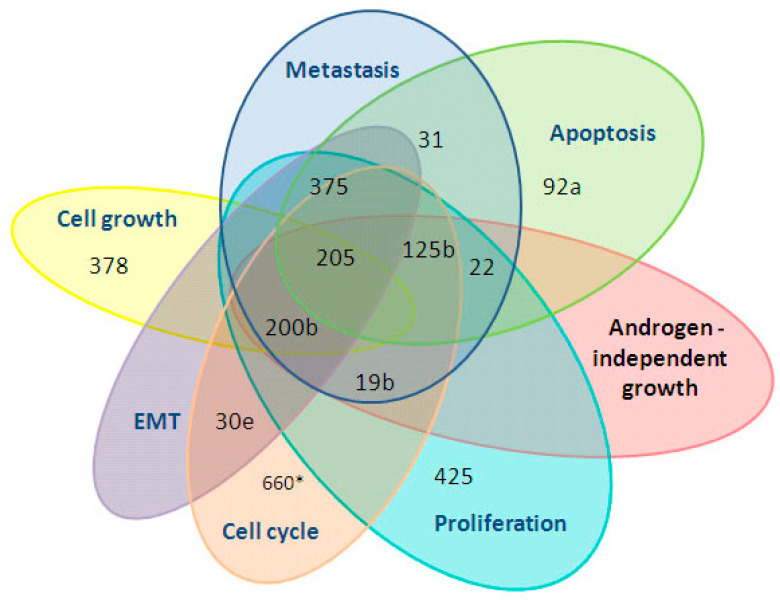
Involvement of the 12 cell-free miRNAs in tumor-related processes. The research papers were available in PubMed and were found by searching the following keywords: prostate, cancer, miR-19b, miR-22, miR-92a, miR-378, miR-425, miR-30e, miR-31, miR-125b, miR-200b, miR-205, miR-375, miR-660. * according to DIANA-mirPath v.3.0.

Eight cell-free miRNA ratios—namely, miR-375/miR-31, miR-375/miR-125b, miR-375/miR-200b, miR-375/miR-205, miR-660/miR-30e, miR-125b/miR-30e, miR-375/miR-30e and miR-660/miR-375—changed after RP in clarified urine, but to a lesser extent than in EVs (Table 2). Only six miRNA ratios changed in blood plasma after RP—namely, miR-125b/miR-30e, miR-375/miR-30e, miR-660/miR-375, miR-660/miR-125b, miR-660/miR-205 and miR-205/miR-30e—but with a maximum ddCt difference of no more than 1.6 (Figure 2 and Figure 3; Table 2).

The most prospective miRNA markers for the evaluation of therapy effectiveness are, obviously, those that maximally changed their ratios (i.e., with maximum ddCt values). Six miRNA ratios with ddCt values of more than 2 were detected in urine EVs and CU (miR-375/miR-30e, miR-660/miR-375, miR-375/miR-31, miR-375/miR-125b, miR-375/miR-200b and miR-375/miR-205). These ratios were based on combinations of seven different miRNAs: miR-30e, miR-31, miR-125b, miR-200b, miR-205, miR-375 and miR-660; all of the miRNA ratios with ddCt values of more than 3 included miR-375. The postsurgical alterations of these ratios were also characterized by a high statistical significance of *p* > 0.05 (and only miR-375/miR-31 with *p* > 0.01 in both urine EVs and CU; Table 2). All ddCt values were lower in CU then in EVs; thus, EVs are the best source of PCs-related miRNA markers among all sources of cell-free miRNAs studied in the present research.

Considering the statistical distribution of the data, the minimum sample size to confirm these differences at the established significance level (5%) and power (80%) did not exceed 16 participants per group, with the exception of the miR-375/miR-30e ratio, whose minimum sample size was 47 participants per group.

The correlation between ddCt and the clinical parameters (i.e., tumor size (via TNM classification of malignant tumors) and Gleason score) and the interval between the surgery and sample collection were examined. A strong reverse correlation between the ddCt of miR-22/miR-378a (k = 0.79) in urine and the Gleason score was revealed. Tumor size (T) positively correlated with the ddCt of miR-425/miR-92a in blood plasma (k = 0.73). The time between surgery and the second sample collection correlated only with the ddCt of miR-425/miR-92a in blood plasma (k = 0.70).

### 3.2. Comparative Analysis of the miRNA Expression in the Biofluids of PCa Patients after RP and in That of Healthy Donors

Table 3 outlines the results of the comparative expression analysis for the miRNA ratios based on dCt in the respective categories. Only statistically significant differences are shown. The expression of 16 miRNA ratios (based on the 12 miRNAs) in urine EVs and four miRNA ratios in blood plasma differed significantly between HDs and PCa patients after RP (Table 3; PCa patients after RP vs. HD).

Most of these ratios had ddCt values of more than 1 (with the exception of miR-425/miR-92a). Analysis of miRNA expression in the biofluids of PCa patients after RP and HDs also confirms that the urine EVs fraction is the most convenient source of miRNA markers. Moreover, a number of miRNA pairs that did not differ between PCa patients before surgery and HDs [15] significantly differed in PCa patients after surgery and HDs. These miRNA pairs list include miR-375/miR-31, miR-375/miR-125b, miR-375/miR-205, miR-660/miR-205 and miR-660/miR-375. The ddCt of these ratios exceeded 3 and were characterized by a *p*-value of <0.01 (Table 3). Seven miRNA expression ratios, altered after RP, satisfied the following conditions: they significantly differed between PCa patients before RP and HDs [15] and recovered to “healthy” phenotype after RP (or even over exceed it). These seven ratios were miR-378/miR-19b, miR-425/miR-19b, miR-125b/miR-30e, miR-200/miR-30e, miR-375/miR-30e, miR-660/miR-30e and miR-205/miR-30e in urine EVs.

Only two of these ratios had ddCt values above 1 and a *p*-value of <0.01, namely, miR-125b/miR-30e and miR-375/miR-30e, representing the most promising targets for evaluation of the effectiveness of therapy.

## 4. Discussion

An analysis of the available literature revealed that the 12 studied miRNAs are involved in such crucial processes of cancerogenesis as proliferation, apoptosis, epithelial mesenchymal transition (EMT), cell growth, cell cycle, metastasis and the development of androgen-independent status, and thus represent a convenient set of predictive tumor recurrence markers (Figure 3).

For all miRNAs, qRT-PCR assays with a working range of 24–38 Ct of PCR were designed. Non-template controls produced no signal or were at least seven cycles apart from the minimum detection limit of a specific template. All reported data were obtained with RNA samples that produced Ct values within the working range of the systems. The spike-in control (cel-miR-39) was detected in all samples at 25 ± 1 Ct.

In this study, we investigated the alteration of the relative expression levels of 12 previously mentioned miRNAs assembled into 31 ratios in three fractions of biologic fluids (i.e., urine EVs, urine clarified urine and blood plasma) from PCa patients before and after RP. Samples of HDs were used as reference. The alteration of the expression of miRNAs in the biofluids of PCa patients after RP has been poorly studied to date, and there are very few studies aimed at comparing miRNAs in the blood of PCa patients before and after RP. In some studies, no significant change was observed in the expression of circulating miRNAs in blood plasma from before to after RP (2 months after RP: [19,20]; 5–6 months after RP: [19]). According to other studies, RP significantly alters miRNA blood levels. For example, a reduction in the expression of miR-16, miR-26a, miR-195 [7], miR-93 and miR-221 [9] and an elevation in the expression of miR-21 and miR-141 in the blood plasma of PCa patients after RP have been reported [8]. Thus, there is no common and well-approved standpoint regarding the influence of RP on the expression of cell-free miRNAs and their usefulness for predicting therapy efficacy.

Here, we demonstrated an influence of RP on the expression of 19 miRNA ratios (Figure 2 and Figure 3). Urine EVs were the source of most the differentially expressed miRNAs, which indicates that urine EVs better reflect the state of the donor and are the most promising source of diagnostic and prognostic markers for PCa, at least of those under study. This is in line with our previous data [13,15,16]. Some of the miRNA ratios in PCa patients after RP caught up with the expression levels of HDs, and some of them moved toward reconstitution of HD expression levels, while others differed by modulus (up- or downregulated) from HDs, even stronger than the pre-surgical levels in PCa patients. Several miRNA ratios changed after RP in two or three biofluids fractions simultaneously (Table 2; Figure 3). Not surprisingly, these changes in urine EVs and CU were unidirectional. At the same time, four miRNA ratios changed their expression in blood plasma in the opposite direction compared to that of urine EVs and CU (i.e., miR-660/miR-205, miR-125b/miR-30e, miR-375/miR-30e and miR-660/miR-375). The different ways in which the miRNAs leave cells, determining their lifetime in extracellular medium [21,22], and the different efficacies of the miRNAs released into biologic liquids by different tissues are obviously responsible for this phenomenon. Moreover, the alterations of miRNA expression in different biofluids may reflect different pathophysiological processes taking place at a particular moment in the tumor process [23]. Of course, a patient’s oncological status is not the only cause of changes in the expression of extracellular miRNAs after RP. Tissue resection, inflammation, reaction to anesthesia (e.g., neurotoxicity), infections, cardiological and urological complications, tissue proliferation and regeneration, as well as direct absence of the prostate gland can also cause alterations in miRNA expression. The duration of their impact on patients can differ a lot. All of these processes should be taken into account when analyzing postoperative miRNA alterations. Since the duration after RP in the present study was only six days, the impact of all of listed factors may have taken place. Egidi et al. showed that both miR-21 and miR-141 were significantly increased by the 5th postoperative day, after which a gradual return to the preoperative levels was recorded. Therefore, the authors suggested that miR-21 and miR-141 change after RP due to postsurgical inflammatory processes and do not seem to be connected with the patient’s oncological status [8]. Individual surgery tolerance and the time course of inflammation lead to questions regarding the optimal patient sampling process. We collected the samples at the time of hospital discharge because it is the most convenient time for both the patient and the physician in order to justify the treatment strategy. Moreover, the collection of samples at discharge allows to establish a level baseline health status from which it is possible to assess the level of miRNAs in the biologic fluids of donors with a stable state. Obviously, sequential tests every few months are necessary to specify a prognosis and to monitor both the patient’s recovery after surgery and the state of the tumor.

The similarity between tumor growth and tissue reparation represent another problem in data analysis. Indeed, many oncogenic and tumor-suppressive miRNAs affect tumor growth by inducing or inhibiting cell proliferation, respectively [24,25]. Thus, after surgery, it seems logical to expect an alteration in the expression of such miRNAs that were previously produced by PCa cells and the tumor microenvironment. At the same time, postsurgical inflammation at the last stage includes the processes of proliferation during wound healing, which can involve, among others, the same regulatory miRNAs that induce and inhibit proliferation. As a result, postsurgical alterations in the expression of these miRNAs are leveled. Similarly, simultaneous multidirectional changes in the expression of miRNAs associated with cell migration, cell growth, cell cycle regulation, etc. can be observed. Moreover, changes in miRNA expression that develop as a result of postoperative inflammation may prevail over those associated with a change in cancer status. On the other hand, being over 50 years old is a risk factor not only for PCa, but also for the development of other diseases, for example, cardiovascular pathologies, the course of which can be aggravated by surgical intervention using anesthesia—in particular, RP [26]. Some of the miRNAs from our panel also act as regulators of the cardiovascular system. For example, miR-19b regulates cardiomyocyte apoptosis and miR-22 regulates endothelial cell proliferation, while miR-378a (miRBase, TargetScanHuman) and miR-200 [27] stimulate and miR-205 [28] inhibits angiogenesis. Thus, the elements of systems, including the cardiovascular system, can affect the postoperative expression of these miRNAs. Nevertheless, miRNAs strongly involved in PCa development were initially selected for present work; this indicates that the alteration of miRNA expression described in present article are mostly due to the changes in oncological status of patient rather than to other reasons.

**Figure 4:**
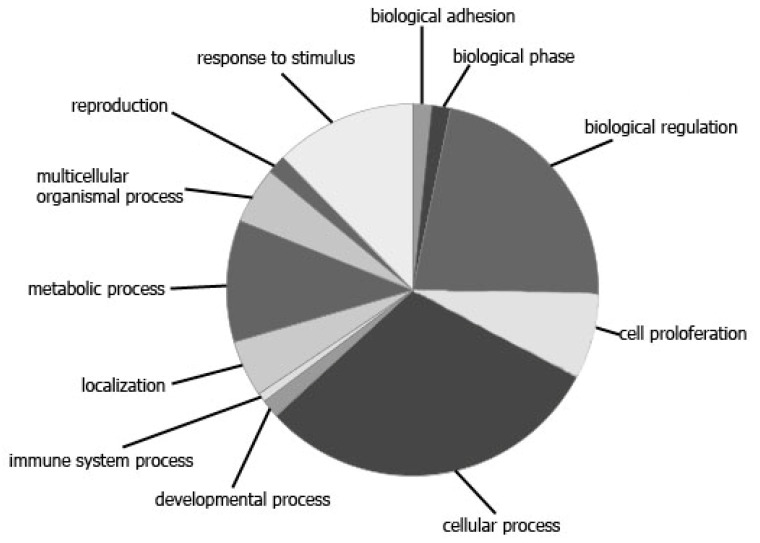
Target genes for miR-660, miR-30e, miR-125b, miR-205, miR-375, miR-378, miR-19b and miR-425, which are involved in prostate cancerogenesis. PANTHER data: GO-Slim Biologic Process.

In light of the foregoing, the miRNAs whose expression level after RP became closer to their ratios in HDs are of the greatest interest in terms of the search for diagnostic and prognostic markers and miRNAs with a crucial role in the pathogenesis of PCa. Such miRNAs are most likely to be specific for changes in the patient’s oncological status than for other processes accompanying RP. Of the 19 miRNA ratios that significantly changed after RP, seven satisfied the following conditions: These ratios significantly differed between PCa patients before RP and HDs and changed in the biological fluids of PCa patients after RP in the direction of the expression levels of HDs (or even caught up with them). These seven ratios were miR-378/miR-19b, miR-425/miR-19b, miR-125b/miR-30e, miR-200/miR-30e, miR-375/miR-30e, miR-660/miR-30e and miR-205/miR-30e in urine EVs, based on a combination of nine miRNAs (i.e., miR-30e, miR-125b, miR-200b, miR-205, miR-375, miR-660, miR-19b, miR-378 and miR-425). Most of these miRNAs, namely, miR-125b, miR-200b, miR-205, miR-375, miR-19b and miR-425, are involved in the regulation of the proliferation of PCa cells (Figure 1; [29,30,31,32,33,34]). In addition, the miRNA ratios miR-125b/miR-30e, miR-200b/miR-30e and miR-660/miR-30e in urine EVs distinguish patients with PCa from HDs and patients with BPH with 100% sensitivity and 100% specificity [15]. To confirm the significance of the selected miRNAs as potential markers of therapy efficacy, we conducted bioinformatics analysis using available databases. According to DIANA-mirPath v.3.0, all of these miRNAs are involved both in PCa development and in cell cycle regulation. They also take part in the p53, mTOR (except miR-660), TGF-beta (except miR-205), Wnt (except miR-205), HIF-1, MAPK, TNF, ERBB, Rap1, AMPK and Foxo signaling pathways, which are crucial for cancerogenesis. Forty-six genes that are involved in the development of PCa and that are potential targets for this miRNA set were found, with a statistical significance of *p* < 0.05 (DIANA-mirPath v.3.0). The involvement of these genes in various biologic processes in accordance with the PANTHER database are presented at Figure 4.

**Figure 5:**
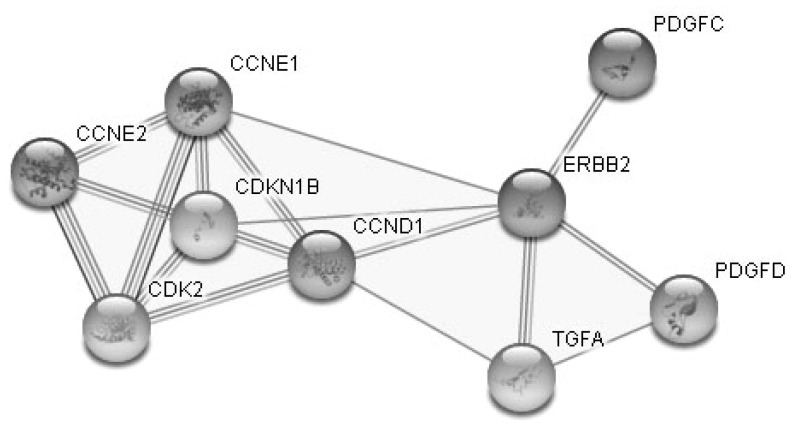
STRING data: Interactional network of the nine genes responsible for “cellular proliferation”.

According to the PANTHER database they mainly belong to the genes responsible for such biologic processes as “biologic regulation”, “cellular process”, “response to stimulus” and “metabolic process” (Figure 4). Figure 5 demonstrates interconnections of nine genes regulating cell proliferation (STRING database).

The proteins encoded by these genes regulate the phosphorylation process; they functionally fall into cell cycle regulators and growth factors. Two of these genes—CCNE1 and CCNE2—also play a part in reproduction processes (PANTHER data).

**Figure 6:**
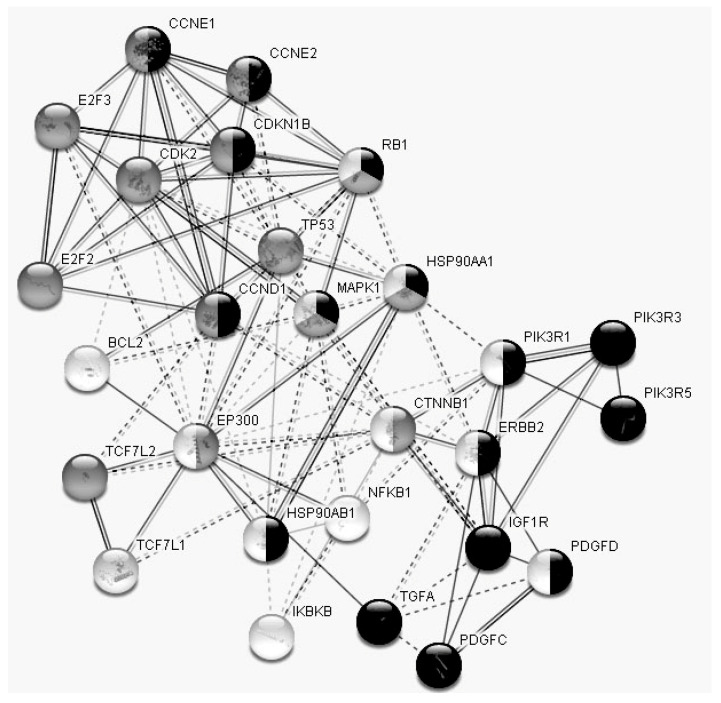
STRING data: Interactional network of the 27 genes responsible for “biologic regulation”. Genes involved in cell cycle regulation indicated in gray, in the regulation of the immune system process in light gray and in kinase activity regulation in dark gray.

According to PANTHER, 27 of the studied genes play a part in “biologic regulation”: HSP90AB1, PIK3R1, TP53, IGF1R, TCF7L1, MAPK1, E2F3, NFKB1, PIK3R5, CTNNB1, CDKN1B, CDK2, PDGFC, ERBB2, BCL2, HSP90AA1, CCNE1, CCNE2, TCF7L2, TGFA, RB1, PIK3R3, PDGFD, E2F2, CCND1, EP300 and IKBKB (Figure 4). These genes form a rather complicated interaction network (Figure 6), as do the six genes responsible for “cellular proliferation”—proteins encoded by these genes have more interactions among themselves than would be expected for a random set of proteins of a similar size, drawn from the genome. Such enrichment indicates that the proteins are at least partially biologically connected as a group. As has emerged, all of these 27 genes functionally fall into three main groups (Figure 6), namely, those involved in cycle regulation, those in immune system processes and those in kinase activity regulation.

Obviously, all these groups are crucial for PCa. The proteins encoded by these genes are mainly involved in the PI3K–Akt signaling pathway (20 gene products); many of them are also part of the MAPK, HIF1, FoxO, Rap1 (7–9 gene products), ERBB, TNF, p53, mTOR, Wnt (5–6 gene products) pathways and a few of them (3–4 gene products) are also engaged in the Vegf and AMPK signaling pathways (String). Thus, the analysis of various databases revealed that the selected miRNAs (i.e., miR-660, miR-30e, miR-125b, miR-205, miR-375, miR-19b and miR-425) are of crucial importance for PCa development and are involved in the regulation of this process at different stages. This is additional evidence that selected miRNAs can indeed be further explored as potential markers of treatment efficacy, as well as potential targets for new therapeutic agents. For example, IGF-1R is among the target genes of the studied miRNA panel (Figure 6). Cixutumumab, a monoclonal antibody directed against IGF-1R, has passed Phase II clinical trials for PCa [35].

**Figure 7:**
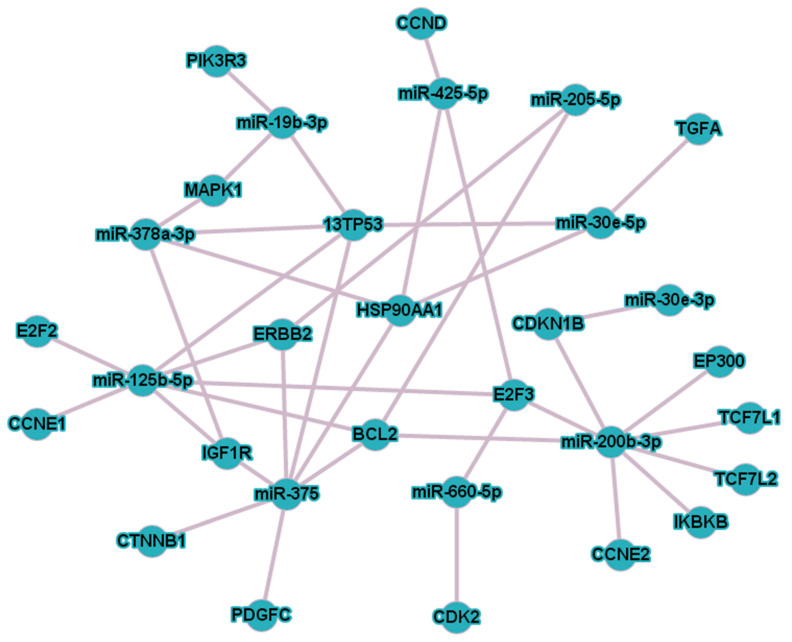
The altered expression of the miRNAs after RP and their target genes responsible for “cellular proliferation” and “biologic regulation” (according to the PANTHER database) visualized using the miRnet database.

The analyzed miRNAs and their target genes responsible for “cellular proliferation” and “biologic regulation” were visualized using the miRnet database (https://www.mirnet.ca/; Figure 7). This allowed to rapidly analyze which of the selected genes is targeted by the studied miRNAs. Interactions of 10 miRNAs and 21 genes were shown by the miRnet database. The most valuable miRNAs and genes from the network are those with the maximum number of associations with the others. In the analyzed panel of miRNAs, the most valuable were miR-200b (8 targets), miR-375 (7 targets), miR-125b (7 targets) and miR-30e (4 targets).

The statistical analysis revealed that the minimum sample size to confirm the alteration of these miRNA ratios at high significance and power did not exceed 47 participants per group. These data indicate the minimum sample size for future verification research and, of course, it should be adjusted in accordance with the new data obtained from an independent sample but can be used unchanged if the variance of the samples matches. In this study, seven of the analyzed miRNA ratios changed their dCt values toward the values of HDs. However, only two of these ratios had ddCt values above 1 and a *p*-value of <0.01: miR-125b/miR-30e and miR-375/miR-30e. Moreover, these two ratios were among the tree miRNA ratios that changed their expression after RP in all of the studied biofluids fractions. All of these facts confirm that miR-125b/miR-30e and miR-375/miR-30e are the most promising miRNA ratios, from the biologic and clinical perspectives, for further research as markers of the effectiveness of therapy. Furthermore, the observed alterations in the miRNA ratios are consistent with the biologic role of these miRNAs in PCa development. The increased miR-125b/miR-30e and miR-375/miR-30e dCt ratios after RP indicate that the expression of miR-30e increased and/or that of miR-125b and miR-375 decreased after RP. This is not unexpected, since miR-125b and miR-375 are known as being oncogenic and are usually elevated in PCa (miR-125b regulates cell cycle, proliferation and apoptosis, while miR-375 regulates proliferation, metastasis and epithelial mesenchymal transition in PCa [36,37,38,39]), while miR-30e is known to be an oncosuppressor and to inhibit PCa proliferation and tumor growth [40,41]. Moreover, all of these three miRNAs were assessed as valuable in the miRNA–gene network analyzed above (Figure 7), indicating their key role among the miRNAs that changed their expression after RP. At the same time, other pairs of miRNAs should not be discounted, because with an increase of time after surgery, their ddCt values can significantly rise.

The differences in several miRNA ratios between PCa patients after RP and HD were greater than between PCa patients before and after RP. Moreover, the magnitude of the ratio of before and after RP did not correspond to that between PCa patients and HDs. The list of these miRNA ratios includes miR-375/miR-31, miR-375/miR-125b, miR-375/miR-200b and miR-660/miR-375. The primary RT-qPCR data demonstrate that this phenomenon is concerned with a significant downregulation of miR-375 expression after RP. This miRNA is known to be frequently overexpressed in PCa, as a negative regulator of apoptosis and a positive regulator of proliferation, metastasis and epithelial mesenchymal transition in PCa (Figure 1). High miR-375–3p levels are also associated with a more advanced pathologic stage and developed metastasis [42]. In our previous comparative study of second stage PCa patients and HDs, only a difference in the miR-375/miR-30e ratio in urine EVs was found [15], thus indicating the moderate overexpression of miR-375. However, a significant downregulation of miR-375 after RP was observed. The processes regulated by miR-31, miR-125b, miR-200b and miR-660 are related, to some extent, with tumor stage and are changed, to a lesser degree, by tumor resection. In any case, these ratios indicate that in PCa patients after RP, their biofluids are not equivalent to that of HDs. There are a vast number of processes that could possibly contribute to this phenomenon, among which are inflammation, scarring, metastases, individual sensitivity to surgical intervention and drugs, characteristics of the healing process and the absence of prostate. The long-term study of the dynamics of such miRNA ratios after RP is prospective in terms of the search for prognostic biomarkers as soon as they distinguish patients after RP and HDs. The expression of some miRNAs in the biofluids of PCa patients after RP could differ from that of HDs even if when they are in full remission. In the future, it is necessary to investigate whether these ratios in patients with PCa after RP reach the level of that in HDs and if so, when this occurs, as well as to determine what processes underlie changes in these ratios.

Investigations of postsurgical miRNA expression are challenged not only by the abovementioned surgical and postsurgical processes, but also by the nature of PCa as a heterogeneous disease. Different characteristics of PCa, as well as features and consequences of the operational process, observed only in some donors, can increase the variability of miRNA expression. This variability impedes data analysis and subsequent identification of potential markers of treatment efficacy or relapse. For example, some of the studied miRNA ratios were characterized by a greater variability compared to the others. These include miR-425/miR-19b in CU and miR-425/miR-92a, miR-19b/miR-92a, miR-22/miR-92a and miR-378a/miR-92a in urine EVs. Remarkably, all of these ratios feature either miR-19b or miR-92a, which are both part of the miR-17-92a cluster. The miR-17–92a cluster has oncogenic properties due to its involvement in the regulation of cell survival, proliferation, differentiation and the cell cycle. Stimulation of this cluster’s hyperexpression in PCa cell line DU-145 leads to an increase in proliferative, migratory and invasive activities [43]. Detailed analysis of these ratios indicates that the postsurgical alterations of the miR-425/miR-92a and miR-378a/miR-92a ratios in two donors were in contrast with overall trend, which could be related to the lack of perineural or perivascular invasion in these patients in contrast to all other PCa patients. This indicates that these two donors may have PCa with attenuated cell migration and invasive activities (related to the miR-17-92a cluster) in comparison to the other patients, who had perineural and/or perivascular invasion. A correlation between the Gleason score and miRNA expression represents another example. It is known that the expression of some miRNAs that are involved in cancerogenesis is correlated with the Gleason score and tumor size [7,44,45,46,47]. In present study, a reliable direct correlation was found between Gleason score and the difference before and postoperative ddCt of miR-22/miR-378a in CU. Thus, the higher the Gleason score and, accordingly, the less differentiated the tumor cells, the greater the increase in the miRNA ratio after RP. This correlation indicates the possibility of multidirectional changes in miRNA expression after RP according to the degree of tumor differentiation. This fact provides some tips for explaining the variation in the data. Indeed, tumor stage and differentiation status, as well as clinical parameters, differ between patients, thus increasing the variability and complexity of the selection of miRNA predictors of PCa, which impedes any further development. Thus, to conduct an analysis of these miRNA ratios, donors should be divided into groups with the same Gleason score, at least in the first stages of marker verification. We also discovered a positive correlation between tumor size (via TNM) and the following miRNA ratios: miR-205/miR-200b in urine EVs, miR-205/miR-31 in CU, miR-425/miR-92a in blood plasma after RP and also a pre- and post-surgical difference in ddCt of the miR-425/miR-92a ratio in blood plasma. To conduct an analysis of these miRNA ratios, donors should be divided into groups with similar tumor sizes (via TNM). However, the contrast in the alterations of the miRNA ratios after RP could be helpful if they occur in patients with and without relapse.

The variations in time between RP and patient discharge, i.e., the moment of the recollection of biologic fluids, can also lead to an increased variability of the miRNA ratios. On one hand, the collection of samples at discharge levels patients, forming a baseline that makes it possible to assess the level of miRNAs in the biologic fluids of donors with an already stable state, which is simple and can provide preliminary information on surgery efficacy and the patient’s state in the near future. On the other hand, the dynamics of some miRNA ratios may depend more on the time elapsed since the removal of the prostate gland. Nevertheless, a longer follow-up time after RP and careful data analysis, including comparison with HDs and patients with non-oncological diseases of the prostate, are warranted to investigate the possible influence of RP on circulating miRNAs.

## 5. Conclusions

In this study, we described the significant alteration of 19 miRNA ratios, based on a combination of 10 different miRNAs, in the biologic fluids of PCa patients after RP. The miRNA ratios with the highest potential for further investigation as therapy effectiveness and relapse markers were miR-125b/miR-30e and miR-375/miR-30e, followed by miR-378/miR-19b, miR-425/miR-19b, miR-200b/miR-30e, miR-660/miR-30e and miR-205/miR-30e. Furthermore, urine EVs showed the highest potential as a source of markers. The obtained results reflect the biology of PCa tumor development and present a preliminary set of new markers for the assessment of PCa therapy effectiveness and for the prognosis of the disease course.

Taking into account the ddCt values, the statistical significance of the data obtained and the miRNAs involved in the molecular mechanisms of PCa progression, verification of miR-125b/miR-30e and miR-375/miR-30e in urine EVs as a marker of PCa therapy effectiveness needs to be carried out. The pilot study data enable to estimate the minimum sample size to confirm the diagnostic efficacy of miRNAs with a high significance level and power. As many as 16 participants per group, with the exception of the miR-375/miR-30e ratio, for which the minimum sample size was 47 participants per group, is necessary to confirm the obtained data. Further verification using a calculated sample size will allow us to study the potential of these miRNAs ratios as predictors of PCa prognosis and RP therapy effectiveness. To conduct an adequate and scientific analysis of the verification data and to develop recommendations for the use of these miRNA markers in clinical practice, more delicate analysis of the expression of miRNAs depending on the PCa stage before and after RP is necessary to elucidate their involvement in PCa biology and to establish criteria for inclusion/exclusion of patients for diagnosis (prognosis) using the proposed miRNAs.

## Figures and Tables

**Table 1:** Overview of the study population.

		PCa	HD
Age	(Mean ± SD)	61.9 ± 5.7	54.8 ± 3.6
	Range	54–71	50–60
Total PSA, ng/mL		8.8 ± 0.95	0.9 ± 0.1
PCa stage	T_1_N_0_M_0_	27.27%	N/A
	T_2_N_0_M_0_	72.72%	
Gleason score	5	9.09%	N/A
	6	27.27%	
	7	63.63%	

**Table 2:** ddCt mean values for differentially expressed miRNA pairs after RP and before RP.

miRNA Ratios	UE		CU		P	
	ddCt	*p*	ddCt	*p*	ddCt	*p*
125b/30e	1.0	**	0.9	^oo^	−1.6	^xx^
375/30e	5.0	**	3.9	^oo^	−1.1	^xx^
660/375	−4.4	**	−3.2	^oo^	0.7	^x^
660/125b	−0.4	**			1.2	^xx^
660/205	1.5	**			1.2	^x^
205/30e	−0.9	*			−1.6	^xx^
375/31	5.0	*	4.5	^o^		
375/125b	4.0	*	2.9	^oo^		
375/200b	4.4	**	4.3	^oo^		
375/205	6.0	**	3.8	^oo^		
660/30e	0.6	*	0.6	^o^		
378/19b	−0.8	*				
425/19b	−0.8	**				
200b/30e	0.6	**				
200b/125b	−0.5	*				
205/31	−1.0	**				
205/125b	−2.0	**				
205/200b	−1.5	**				
125b/31	1.0	**				
Number of differently expressed miRNA pairs	19		8		6	

Wilcoxon paired T-criteria for different samples: ** *p* < 0.01, * *p* < 0.05—urine EVs; ^oo^ *p* < 0.01, ^o^ *p* < 0.05—clarified urine; ^xx^ *p* < 0.01, ^x^ *p* < 0.05—plasma. UE—ddCt in urine EVs; CU—ddCt in clarified urine; P—ddCt in plasma.

**Table 3:** ddCt mean values for differentially expressed miRNA pairs in prostate cancer patients after radical prostatectomy and healthy donors.

miRNARatios	UE		CU	P	
	ddCt	*p*		ddCt	*p*
92a/19b	5.91	***			
22/92a	−5.26	**		1.92	^xx^
22/378a				1.78	^x^
22/425				1.26	^x^
378a/92a	−4.90	***			
425/92a	−5.71	***		0.66	^x^
31/30e	−7.41	***			
125b/30e	−4.98	***			
200b/30e	−7.76	***			
205/30e	−7.95	***			
375/30e	1.53	***			
660/30e	−3.76	***			
375/31	7.42	***			
375/125b	5.00	***			
375/200b	7.78	***			
375/205	7.97	***			
660/205	4.19	**			
660/375	−3.78	***			
Number of differently expressed miRNA pairs	16		0	4	

*** *p* < 0.001, ** *p* < 0.01 in urine EVs; ^xx^ *p* < 0.01, ^x^ *p* < 0.05 in plasma. UE—ddCt in urine EVs; CU—ddCt in clarified urine; P—ddCt in plasma.

## Supplementary Materials

The following are available online at https://www.mdpi.com/2075-4418/10/8/600/s1, Table S1: Sequences of primers and probes used for reverse transcription and TaqMan qPCR, Table S2: ddCt mean values and standard errors for miRNA pairs in biofluids of cancer patients after RP and before RP, biofluids of cancer patients after RP and healthy donors.
