# Supplementary material for: The Influence of Radical Prostatectomy on the Expression of Cell-Free MiRNA
Source: Diagnostics (Basel). 2020 Aug 17;10(8):600. doi: 10.3390/diagnostics10080600 (PMC7460220; doi:10.3390/diagnostics10080600)
Supplement: Supplementary file 1 [file diagnostics-10-00600-s001.pdf]

Table S1. Sequences of primers and probes used for reverse transcription and TaqMan qPCR.

miRNA	Label	Sequence
	Universal Reverse Primer	5′-GTGCAGGGTCCGAGGT-3′
**hsa-miR-19b-3p**	RT	5′-GTCGTATCCAGTGCAGGGTCCGAGGTATTCGCACTGGATACGACTCAGTT-3′
	Forward	5′-CGCTGTGCAAATCCATGCAA-3′
	Probe	5′-(FAM)-GCACTGGATACGACTCAGTT-(BHQ)-3′
**hsa-miR-22**	RT	5′-GTCGTATCCAGTGCAGGGTCCGAGGTATTCGCACTGGATACGACACAGTT-3′
	Forward	5′-AAGCTGCCAGTTGAAG-3′
	Probe	5′-GCACTGGATACGACACAGTT-3′
**hsa-miR-92a**	RT	5′-GTCGTATCCAGTGCAGGGTCCGAGGTATTCGCACTGGATACGACACAGGC-3′
	Forward	5′-CGCTATTGCACTTGTCCCG-3′
	Probe	5′-(FAM)-CGCACTGGATACGACACAGGC-(BHQ)-3′
**hsa-miR-378a-3p**	RT	5′-GTCGTATCCAGTGCAGGGTCCGAGGTATTCGCACTGGATACGACGCCTTC-3′
	Forward	5′-GACTGGACTTGGAGTCA-3′
	Probe	5′-(FAM)-CGCACTGGATACGACAAAGTC-(BHQ)-3′
**hsa-miR-425-5p (miR-425)**	RT	5′-GTCGTATCCAGTGCAGGGTCCGAGGTATTCGCACTGGATACGACTCAACG-3′
	Forward	5′-TAATGACACGATCACTCC-3′
	Probe	5′-(FAM)-CGCACTGGATACGACTCAACG-(BHQ)-3′
**hsa-miR-30e**	RT	5′-GTCGTATCCAGTGCAGGGTCCGAGGTATTCGCACTGGATACGACCTTCCA-3′
	Forward	5′-GCCCTGTAAACATCCTTGAC-3′
	Probe	5′-(FAM)-GCACTGGATACGACCTTCCA-(BHQ)-3′
**hsa-miR-31-5p**	RT	5′-GTCGTATCCAGTGCAGGGTCCGAGGTATTCGCACTGGATACGACAGCTAT-3′
	Forward	5′-CAGGCAAGATGCTGGCA-3′
	Probe	5′-(FAM)-TCGCACTGGATACGACAGCTAT-(BHQ)-3′
**hsa-miR-125b-5p**	RT	5′-GTCGTATCCAGTGCAGGGTCCGAGGTATTCGCACTGGATACGACTCACAA-3′
	Forward	5′-CGTCCCTGAGACCCTAACTT-3′
	Probe	5′-(FAM)-GCACTGGATACGACTCACAA-(BHQ)-3′
**hsa-miR-200b-3p**	RT	5′-GTCGTATCCAGTGCAGGGTCCGAGGTATTCGCACTGGATACGACTCATCA-3′
	Forward	5′-GTAATACTGCCTGGTAATG-3′
	Probe	5′-(FAM)-CGCACTGGATACGACTCATCA-(BHQ)-3′
**hsa-miR-205-5p**	RT	5′-GTCGTATCCAGTGCAGGGTCCGAGGTATTCGCACTGGATACGACCAGACT-3′
	Forward	5′-CCTCCTTCATTCCACCGGA-3′
	Probe	5′-(FAM)-GCACTGGATACGACCAGACT-(BHQ)-3′
**hsa-miR-375**	RT	5′-GTCGTATCCAGTGCAGGGTCCGAGGTATTCGCACTGGATACGACTCACGC-3′
	Forward	5'-GCCCTTTGTTCGTTCGGCTC-3'
	Probe	5′-(FAM)-TCGCACTGGATACGACTCACGC-(BHQ)-3′
**hsa-miR-660-5p**	RT	5′-GTCGTATCCAGTGCAGGGTCCGAGGTATTCGCACTGGATACGACCAACTC-3′
	Forward	5′-CCCATTGCATATCGGAG-3′
	Probe	5′-(FAM)-GCACTGGATACGACCAACTC-(BHQ)-3′
**cel-miR-39-3p**	RT	5′-GTCGTATCCAGTGCAGGGTCCGAGGTATTCGCACTGGATACGACCAAGCT-3′

	Forward	5′-ATTCACCGGGTGTAAATC-3′
	Probe	5′-(FAM)-CACTGGATACGACCAAGCTGA-(BHQ)-3′

Table S2. ddCt mean values and standard errors for miRNA pairs in biofluids of cancer patients after RP and before RP, biofluids of cancer patients after RP and healthy donors.

**miRNA ratios**	**PCa, after vs before RP**		**PCa after RP vs HD**	
	**ddCt mean**	**Standard error**	**ddCt mean**	**Standard error**
	**UE**			
22/19b	-0,16	0,29	0,68	0,26
92a/19b	2,02	1,42	5,95	0,74
378a/19b	-0,85	0,27	1,04	0,27
425/19b	-0,83	0,28	0,23	0,20
22/92a	1,86	1,33	-5,26	0,85
378a/92a	1,58	1,33	-4,90	0,83
425/92a	1,54	1,30	-5,71	0,78
22/378a	0,29	0,26	-0,37	0,33
22/425	0,32	0,24	0,44	0,24
378a/425	0,04	0,26	0,81	0,01
31/30e	0,37	0,33	3,42	0,73
125b/30e	1,04	0,26	-4,98	0,29
200b/30e	0,62	0,38	-7,76	0,80
205/30e	-0,93	0,51	-7,95	0,52
375/30e	5,02	0,31	1,53	0,45
660/30e	0,6	0,20	-3,76	0,37
125b/31	1,04	0,22	1,93	0,73
200b/31	2,94	0,16	1,87	0,56
205/31	-1,05	0,45	-3,04	0,87
375/31	5,04	0,11	7,42	0,79
200b/125b	-0,50	0,22	4,54	0,68
205/125b	-1,51	0,43	-5,45	0,56
375/125b	4,1	0,10	5,00	0,41
660/125b	-0,4	0,26	6,53	0,36
205/200b	-4,75	0,42	-3,13	0,74
375/200b	4,4	0,10	7,78	0,75
660/200b	5,22	0,42	1,28	0,95
375/205	6,01	0,23	7,79	0,54
660/205	1,55	0,40	4,19	0,61
660/375	-4,4	0,26	-3,78	0,51
	CU			
22/19b	0,92	0,41	-0,74	0,38
92a/19b	-0,56	0,32	0,33	0,28
378a/19b	0,62	0,39	-0,50	0,31
425/19b	0,82	0,57	-0,82	0,40
22/92a	0,36	0,49	-0,25	0,47
378a/92a	0,06	0,35	-0,23	0,35

425/92a	0,26	0,33	-0,21	0,32
22/378a	0,30	0,32	-0,02	0,31
22/425	0,10	0,64	-0,05	0,53
378a/425	-0,20	0,54	-0,02	0,51
31/30e	1,05	0,49	1,01	0,36
125b/30e	0,9	0,19	1,68	0,19
200b/30e	1,65	0,81	-1,64	0,24
205/30e	1,85	0,60	-1,14	0,57
375/30e	3,92	0,78	1,02	0,76
660/30e	0,6	0,51	1,07	0,51
125b/31	0,87	0,51	2,64	0,38
200b/31	1,33	0,80	1,38	0,27
205/31	0,55	0,43	-0,80	0,36
375/31	4,55	1,02	0,36	0,96
200b/125b	0,72	0,74	1,00	0,24
205/125b	0,65	0,53	-1,67	0,50
375/125b	2,94	0,68	0,49	0,68
660/125b	0,46	0,49	0,54	0,46
205/200b	1,00	0,68	-1,30	0,42
375/200b	4,34	1,10	0,46	0,81
660/200b	1,70	1,01	0,51	0,62
375/205	3,84	0,86	1,06	0,97
660/205	1,13	0,79	1,23	0,75
660/375	-3,24	0,76	-0,56	0,76
	P			
22/19b	0,24	0,32	0,85	0,30
92a/19b	0,75	0,36	0,88	0,33
378a/19b	-0,71	0,35	-0,78	0,42
425/19b	-0,65	0,26	-0,32	0,25
22/92a	0,99	0,45	1,92	0,42
378a/92a	0,03	0,22	0,10	0,28
425/92a	0,10	0,23	0,66	0,16
22/378a	0,95	0,42	1,78	0,42
22/425	-0,89	0,35	1,26	0,33
378a/425	-0,07	0,23	-0,46	0,27
31/30e	-2,05	1,64	1,57	0,34
125b/30e	-1,64	0,28	1,19	1,23
200b/30e	-1,58	0,48	-1,39	0,45
205/30e	-1,62	0,26	0,50	0,77
375/30e	-1,12	0,30	1,40	0,45
660/30e	1,57	0,20	1,20	0,40
125b/31	5,46	1,75	1,29	0,42
200b/31	-0,75	1,68	-0,08	1,04
205/31	0,62	1,72	0,61	0,25
375/31	4,96	1,72	1,51	0,67
200b/125b	-1,53	0,47	1,68	0,36

205/125b	-0,54	0,34	0,62	0,41
375/125b	1,80	0,34	1,53	0,40
660/125b	1,25	0,23	1,33	1,07
205/200b	-1,60	0,47	-1,28	0,99
375/200b	1,40	0,56	-1,28	1,01
660/200b	1,64	0,48	-0,91	1,27
375/205	-1,00	0,34	-2,44	0,65
660/205	1,21	0,27	0,33	0,39
660/375	0,75	0,36	1,10	0,48

UE: ddCt in urine EVs. CU: ddCt in clarified urine. P: ddCt in plasma.
